# Reducing HIV-related risk and mental health problems through a client-centred psychosocial intervention for vulnerable adolescents in Addis Ababa, Ethiopia

**DOI:** 10.7448/IAS.19.5.20832

**Published:** 2016-07-20

**Authors:** Nrupa Jani, Lung Vu, Lynnette Kay, Kassahun Habtamu, Samuel Kalibala

**Affiliations:** 1 HIVCore/Population Council, Washington, DC, USA; 2 Retrak Ethiopia, Addis Ababa, Ethiopia; 3 School of Psychology, Addis Ababa University, Addis Ababa, Ethiopia

**Keywords:** Ethiopia, mental health, vulnerable adolescents, HIV risk, psychosocial intervention

## Abstract

**Introduction:**

Ethiopia is experiencing an increasingly urban HIV epidemic, alongside a rise in urban adolescent migration. Adolescent migrants are often confronted by unique social challenges, including living in a difficult environment, abuse and mental health problems. These issues can increase adolescents’ vulnerability to HIV and compromise their capacity to protect themselves and others from HIV. We piloted and assessed the effects of a targeted psychosocial intervention to reduce mental health problems and improve HIV-related outcomes among migrant adolescents in Addis Ababa.

**Methods:**

A pre- and post-comparison design was used in a cohort of 576 female and 154 male migrant adolescents aged 15 to 18 years in Addis Ababa receiving services from two service delivery organizations, Biruh Tesfa and Retrak. We implemented a three-month client-centred, counsellor-delivered psychosocial intervention, based on findings from formative research among the same target population, to address participants’ increased vulnerability to HIV. The intervention package comprised individual, group and creative arts therapy counselling sessions. Key outcome indicators included anxiety, depression, aggressive behaviour, attention problems, social problems, knowledge of HIV, safer sex practices and use of sexual health services. Longitudinal data analysis (McNemar test and random effects regression) was used to assess changes over time in key indicators by gender.

**Results:**

For females, aggressive behaviour decreased by 60% (adjusted odds ratio (AOR): 0.4 (0.25 to 0.65)) and any mental health problem decreased by 50% (AOR: 0.5 (0.36 to 0.81)) from baseline to end line. In addition, knowledge of HIV increased by 60% (AOR: 1.6 (1.08 to 2.47)), knowledge of a place to test for HIV increased by 70% (AOR: 1.7 (1.12 to 2.51)) and HIV testing increased by 80% (AOR: 1.8 (1.13 to 2.97)). For males, HIV knowledge increased by 110% (AOR: 2.1 (1.1 to 3.94)), knowledge of a place to test for HIV increased by 290% (AOR: 3.9 (1.02 to 14.9)), HIV testing increased by 630% (AOR: 7.3 (2.6 to 20.7)) and use of sexual health services increased by 220% (AOR: 3.2 (1.62 to 6.27)). We did not find any significant reduction in mental health problems among male adolescents.

**Conclusions:**

Our findings suggest that a psychosocial intervention was associated with increased knowledge and uptake of HIV and sexual health services among both male and female migrant adolescents and with reduced mental health problems among female adolescents. Mental health problems varied significantly for male and female adolescents, suggesting that future interventions should be tailored to address their different needs and would benefit from intensive follow-up efforts.

## Introduction

In 2011, HIV prevalence in Ethiopia was 1.0% for men and 1.9% for women aged 15 to 49 years in the general population [1]. Although Ethiopia has a relatively low national HIV prevalence of 1% among young adults aged 15 to 24 years [2], it is experiencing an increasingly urban and female-centred HIV epidemic [3]. Risk of infection is higher among young people living in urban settings, especially young women (1.7% female; 0.2% male) [2]. Urban HIV prevalence is also higher at 7.7% compared with 0.9% in rural areas [4]. UNAIDS 2013 data estimated that 74% of new adolescent HIV infections in Ethiopia occur among young women [5].

Additionally, Ethiopia is experiencing a surge in rural-to-urban migration, with an increase in young people migrating to urban areas in search of educational and economic advancement opportunities [6]. Young migrants are often confronted by unique social challenges such as: abuse; lack of education, parental guidance and social networks; inadequate housing and access to health services; and unstable employment conditions [3,7]. However, the experiences of young female and male adolescent migrants differ in important ways.

Female migrants are often at a greater disadvantage than males due to lower educational levels and gender norms that allow for subjugation of females [6]. A 2006 population-based survey of 1000 adolescents aged 10 to 19 in Addis Ababa found that nearly 25% of migrant females had moved in order to avoid family-mandated early marriages [3]. It has been shown that 77% of working girls in low-income areas of Addis Ababa were domestic helpers, a job associated with low, if any, pay, extreme social isolation and poor working conditions – all factors that can contribute to an elevated HIV risk [8]. Young women are particularly vulnerable to physical and sexual abuse in their homes, places of work or in transit during migration, increasing their susceptibility to HIV [9].

Male migrant adolescents, too, are vulnerable. A 2009 study found that 29% of male street children in the Merkato area of Addis Ababa had been sexually assaulted [10]. A recent study by Habtamu and Adamu [7] found that among male street adolescents and children, nearly all participants reported having heard about cases of sexual exploitation of their male peers. The authors wrote, “Sexual abuse and exploitation of male migrant adolescents is also one of the emerging social problems affecting the physical, social and psychological wellbeing of children in Addis Ababa” [7]. Furthermore, highly mobile adolescents are particularly vulnerable to sexually transmitted diseases, such as HIV, and circular migration patterns that can facilitate the spread of HIV between high-prevalence urban centres and low-prevalence rural areas [11]. There is also a widespread belief in Ethiopia that men who have sex with men and male sexual abuse are not “Ethiopian” [10], so it can be assumed that male sexual abuse cases go unreported, underreported or misreported.

Resultant negative psychosocial outcomes, such as feelings of guilt, stress, self-blame, anxiety and depression, are common and associated with sexual and HIV-related risks [12,13]. Reasons for increased risk include low self-esteem and self-efficacy, vulnerability to sexual abuse, limited educational opportunities and communication skills, and inability to negotiate safer sex [14]. Nearly 50% of all adult mental health problems begin during adolescence [15], even though they may not be diagnosed until well into adulthood. Research has shown a strong bidirectional link between HIV vulnerability and mental health illnesses [16]. Among adolescents, aggressive discipline, family violence, poor interpersonal relationships and compromised mental health status can increase HIV risk [17], further supporting the need for mental health interventions to halt the spread of the virus.

To date, there is no evidence about the effects of targeted psychosocial interventions on mental health and HIV-related outcomes of young migrant adolescents in Ethiopia. This is the first operations research study aiming to pilot and assess the effects of a targeted psychosocial intervention among this target population in Ethiopia. The intervention was designed to reduce mental health problems, such as anxiety and aggressive behaviour, and to improve HIV-related outcomes, including knowledge of HIV, safer sex practices and use of sexual and reproductive health (SRH) services. The findings will serve as an evidence base for future interventions targeting migrant and vulnerable adolescents in Ethiopia and similar contexts.

## Methods

### Study design and sample size

The study used a pre- and post-comparison design with the same individuals being followed up over time. Participants included consenting male and female adolescents aged 15 to 18 years in Addis Ababa, Ethiopia, receiving services for the previous three months from two service delivery organizations, Biruh Tesfa and Retrak. Biruh Tesfa works with female migrant adolescents who are predominantly employed as domestic workers, whereas Retrak works with male migrant adolescents who are often engaged in street labour activities, such as petty trade and as bus station porters. Based on client volume at both organizations, we recruited all eligible youth who consented to participate in the study. We recruited a total of 576 eligible females at Biruh Tesfa and 154 eligible males at Retrak in June and July 2013. Following the three-month intervention period, we re-interviewed 315 female participants (56.6%) and 102 male participants (68.5%) at end line.

### Description of the intervention

We piloted a client-centred, psychosocial counselling intervention package that was delivered by study counsellors for three months. The intervention package comprised various counselling modalities, including individual, group and creative therapies, such as music, art and drama. All enrolled participants received a minimum of one initial, individual counselling session. Afterwards, each participant was assessed again by his/her counsellor and referred for further group or individual counselling. If after the first session the counsellor deemed that the participant would benefit from further counselling to discuss deeper rooted emotional issues, then he/she was referred for group counselling in the format of art, music or drama therapy.

Counsellors maintained a counselling record book to document issues discussed and enable follow-up in subsequent sessions, as needed. This package of intervention components was modelled on problem-solving therapy, which has been shown to be effective in helping young people deal with a wide range of difficulties and problems that occur in everyday living [19–21].

Counsellors were trained using a standard training curriculum developed by the study team with inputs from both service delivery organizations. Counselling training topics included: adolescent health and development; psychological wellbeing and mental health problems; factors increasing vulnerability of marginalized adolescents; concepts of and ethical issues in counselling; counselling theories, skills and processes; group counselling; and creative therapies and music and drama therapies.

The five-day counsellor training included a practicum day and a one-day refresher training held midway (1.5 months) through the intervention. Counsellors used a client-centred approach, addressing the following: 1) main issues brought forth by the client; 2) possible options and solutions to address the issues and the pros and cons of each option; and 3) plan of action selected by the client to address the problem. Counsellors were also trained to cover topics of sexual health and HIV and AIDS (knowledge, risks and prevention strategies), alcohol and drug abuse, and previous or current experience with violence.

### Measurements

A study expert committee examined four mental-health screening tools and considered the applicability of these tools in Ethiopia based on available guidance and ease of administration, scoring, results interpretation, applicability of the tool for the target age group, and adequate coverage of a range of mental health conditions. The Youth Self-Report (YSR) was chosen as the most appropriate tool for use in this context. This tool went through a systematic process of cultural adaptation and validation. The adapted version, translated into Amharic, was used to measure mental health problems among study participants. Details of the process for adapting the scale, testing for reliability and validity and determining the cutoff points are described elsewhere [22]. The following is a summary of the adaptation and validation process. The YSR identifies eight syndromes approximating the diagnoses from the *Diagnostic and Statistical Manual of Mental Disorders*, *4th Edition* [23]. We chose four syndromes for use in this study based on their prevalence within the sample during the adaptation process (anxious/depressed, social problems, attention problems and aggressive behaviour).

We measured anxiety using a 13-item scale, including such statements as “I am afraid of certain situations or bad things” or “I worry a lot.” We measured social problems using an 11-item scale, including such statements as “I don't get along with others” or “I am not liked by other kids.” Attention problems were measured using a nine-item scale, including such statements as “I have trouble concentrating or paying attention” and “I act without thinking.” Aggressive behaviour was measured using a 17-item scale, including such statements as “I am mean to others” and “I scream a lot.”

Each statement has three answer options: 0=true, 1=somewhat true, 2=very true. During the adaptation, scores from each scale were aggregated and internal validity was assessed (alpha of 0.70 and above). A receiver operating characteristic (ROC) analysis was conducted to determine the area under the curve. The ROC analysis determined the cutoff scores for anxiety problems at 3.5, social problems at 2.5, attention problems at 3.5 and aggressive behaviour at 2.5. The adapted tool was administered verbally to study participants due to low literacy levels.

We measured HIV-related indicators using a validated behavioural survey adapted from the Demographic Health Survey to collect information about key demographic characteristics, experience of abuse, knowledge of HIV, knowledge of a place to test for HIV, perceived risk of HIV infection and use of SRH services. HIV knowledge was measured using the five-item scale from UNAIDS, which has been used and validated in HIV Indicator Surveys and Demographic and Health Surveys [24]. The scale is comprised of five questions about HIV transmission and prevention knowledge: 1) Can people reduce HIV risk by having one sexual partner?; 2) Can people get HIV from mosquito bites?; 3) Can people reduce HIV by using a condom?; 4) Can people get AIDS as a result of witchcraft?; and 5) Is it possible for a healthy-looking person to have HIV?. If participants answered all five questions correctly, they were determined to have “comprehensive knowledge” of HIV.

### Data analysis

The analysis was stratified by gender. We included data from 315 female participants and 102 male participants, who completed both baseline and end line surveys, to identify changes in key outcome indicators over time. We used analysis techniques applied to longitudinal data (or correlated data). We used the McNemar test at the bivariate level to assess changes in key indicators over time. At the multivariate level we used multiple logistic regression (random effects), adjusting for key socio-economic characteristics. The rationale behind the random effects model is that, unlike the fixed effects model, the variation across individuals is assumed to be random and uncorrelated with the independent variables included in the model. To decide between fixed or random effects, we performed a Hausman test, where the null hypothesis is that the preferred model is random effects *versus* the alternative, fixed effects [25]. Our analyses indicated that random effects regression should be chosen. All analyses were performed in Stata (StataCorp LP, 4905 Lakeway Drive College Station, Texas 77845-4512, USA. version 13).

### Ethical considerations

The study was approved by the Population Council Institutional Review Board and the Addis Ababa City Administration Health Bureau. Research activities involving adolescents followed guidance outlined in *Ethical Approaches to Gathering Information from Children and Adolescents in International Settings: Guidelines and Resources* [26]. Young people living outside of parental care are considered *emancipated minors*, defined as living independently of their parents and having the right to make decisions about receiving services without necessitating parental or guardian consent. All participants provided written informed consent.

## Results

### Sample characteristics at baseline

Table 1 presents the characteristics of the study population at baseline. More than half of the sample (55.7%) had attained one to five years of schooling, and 42% had attained six to eleven years of schooling.

**Table 1:** Characteristics of the study population at baseline

	Females (*N=*557) % (*n*)	Males (*N=*149) % (*n*)
Age		
15–16 years old	62.8 (350)	47.3 (70)
17–18 years old	37.2 (227)	52.7 (78)
Education		
1–5 years of schooling	54.0 (301)	57.7 (86)
6–11 years of schooling	46.0 (256)	42.3 (63)
Literacy level		
Unable to read at all	28.8 (156)	29.4 (35)
Read part of the sentence	20.5 (111)	14.3 (17)
Read whole sentence	50.7 (275)	56.3 (67)
Religion		
Orthodox	77.7 (433)	55.7 (83)
Muslim	15.1 (84)	30.9 (46)
Protestant	7.2 (40)	13.4 (20)
Currently living with relative or family		
Yes	70.7 (394)	0.0 (0)
No	29.3 (163)	149.0 (100)
Place lived previously		
Addis Ababa	19.0 (106)	11.6 (17)
Other city	2.7 (15)	14.9 (22)
Small town	10.4 (58)	36.1 (53)
Rural village	67.9 (378)	37.4 (55)
Keep in touch with family		
Yes	87.4 (485)	34.2 (51)
No	12.6 (70)	65.8 (98)
Ever had sex		
Yes	0.6 (3)	6.7 (10)
No	99.4 (496)	93.3 (139)
Mental health outcome		
Had social problem	15.8 (88)	49.0 (73)
Had attention problem	10.0 (56)	36.2 (54)
Had anxiety/depression problem	21.5 (120)	47.0 (70)
Had aggressive behaviour	23.0 (128)	43.2 (45/104)*
Had any mental health problem	37.3 (208)	80.8 (101/125)**

*Notes:* Values of *n* in cells may not add up to total population due to missing values.

* *N=*104

** *N=*125 due to missing values.

Nearly three-quarters of the male sample (70.6%) could read part or the entirety of a sentence in a local language, whereas 29.4% could not read at all. About 39% of males had temporary employment, 37.9% were self-employed and 15.8% were unemployed. About one-third of males had migrated from a rural village (37.4%) or from a small town (36.1%), and the rest had migrated from another urban town. More than half of the young men (55.7%) were Orthodox Christian, 30.9% were Muslim and 13.4% were Protestant. Very few young men in the sample reported being sexually active (7%). With regard to mental health status at baseline, 49% had social problems, 36.2% had attention problems, 47% had anxiety/depression and 43.2% had aggressive behaviour.

About 54% of the female sample had one to five years of schooling and 46% had six to eleven years of schooling. Approximately 70% of the female sample could read part of or an entire sentence in a local language. The majority of young women (64.4%) were permanently employed. Only 15.3% were temporarily employed and 11% were unemployed. More than three-quarters of young women (77.7%) were Orthodox Christian, 15.1% were Muslim and 7.2% were Protestant. About two-thirds of the sample reported having migrated to Addis Ababa from a rural area. Only three young women reported being sexually active during the past 12 months (0.6%). With regard to mental health outcomes, 15.8% had social problems, 10% had attention problems, 21.5% had anxiety/depression and 23% had aggressive behaviour.

### Characteristics of participants lost to follow-up at end line

Overall, those lost to follow-up (LFU) and those who completed the study were comparable with regard to key characteristics. However, we experienced greater LFU among females in the 17 to 18 age group and who had lower levels of literacy, and among males who had higher literacy levels and fewer anxiety problems (data not shown).

### Effects of the intervention on key mental health and HIV-related outcomes

For females, bivariate analyses (Table 2, McNemar test) suggest that the intervention was associated with reduced attention problems and aggressive behaviour and the combined mental health index, “having any one of the four mental health problems.” In addition, the intervention was associated with increased knowledge of HIV prevention, knowing a place to test for HIV and having ever tested for HIV. Multivariate logistic regression adjusting for age, education and religion showed that the intervention was associated with a 60% reduction in aggressive behaviour (adjusted odds ratio (AOR): 0.4 (0.25 to 0.65)), a 50% reduction in “any of the four mental health problems” (AOR: 0.5 (0.36 to 0.81)), a 60% increase in comprehensive knowledge of HIV (AOR: 1.6 (1.08 to 2.47)), a 70% increase in knowing a place to test for HIV (AOR: 1.7 (1.12 to 2.51)), and a 80% increase in “ever tested for HIV” (AOR: 1.8 (1.13 to 2.97)).

**Table 2:** Effects of the intervention on mental health and HIV-related outcomes (bivariate analysis)

	Females (*N=*315)			Males (*N=*102)		
		
Variable	Baseline % (*n*)	End line % (*n*)	*p*	Baseline % (*n*)	End line % (*n*)	*p*
Mental health outcomes						
Had anxiety problem	20.0 (63)	14.9 (47)	0.07	54.0 (55)	52.0 (53)	0.72
Had social problem	13.3 (42)	8.9 (28)	0.07	51.0 (53)	52.0 (52)	0.87
Had attention problem	8.6 (27)	4.8 (15)	0.05	37.3 (38)	38.2 (39)	0.88
Had aggressive behaviour	24.4 (77)	13.0 (41)	0.0001	53.2 (32)	59.5 (49)	0.47
Had any mental health problem	34.9 (110)	15.1 (79)	0.0035	75.5 (77)	73.5 (75)	0.71
HIV-related outcomes						
Had comprehensive knowledge of HIV	16.8 (53)	24.4 (77)	0.014	20.6 (21)	34.3 (35)	0.04
Perceived HIV risk (low vs. medium+high)	17.1 (54)	14.3 (45)	0.29	23.5 (24)	27.5 (28)	0.51
Knew a place to test for HIV	68.6 (216)	77.2 (243)	0.009	88.2 (90)	95.1 (97)	0.05
Ever tested for HIV	29.8 (86)	36.1 (104)	0.000	45.1 (46)	69.6 (71)	<0.000
Use of SRH service (past 3 months)	48.9 (154)	45.4 (143)	0.31	31.4 (32)	54.9 (56)	0.0004

*Notes: p* value corresponds to McNemar test; the analyses were performed on those who had complete baseline and end line data. SRH, sexual and reproductive health.

For male adolescents, bivariate analyses suggest that the intervention was associated with an increase in HIV knowledge, knowledge of a place to test for HIV, having ever tested for HIV and seeking SRH services. Multivariate analyses, adjusting for age, education and religion, as presented in Table 3, show that the odds of having comprehensive knowledge of HIV increased by 110% (AOR: 2.1 (1.10 to 3.94)) compared with baseline, knowing a place to test for HIV increased by 290% (AOR: 3.9 (1.02 to 14.9)), having tested for HIV increased by 630% (AOR: 7.3 (2.6 to 20.7)) and use of SRH services increased by 220% (AOR: 3.2 (1.62 to 6.27)). The intervention was not associated with any changes in the five key mental health indicators for males.

**Table 3:** Effects on mental health and HIV-related outcomes (multivariate analysis)

	Females	Males
	
	End line AOR (95% CI)	End line AOR (95% CI)
Mental health outcome		
** **Had anxiety problem	0.7 (0.41–1.06)	0.9 (0.43–1.76)
** **Had social problem	0.6 (0.36–1.07)	1.1 (0.56–2.1)
** **Had attention problem	0.6 (0.30–1.24)	1.0 (0.53–1.78)
** **Had aggressive behaviour	0.4 (0.25–0.65)***	1.4 (0.72–2.89)
** **Had any mental health problem	0.5 (0.36–0.81)**	0.5 (0.22–1.29)
HIV-related outcome		
** **Had comprehensive knowledge of HIV	1.6 (1.08–2.47)*	2.1 (1.10–3.94)*
** **Perceived HIV risk (low *vs*. medium+high)	0.8 (0.47–1.21)	1.2 (0.62–2.30)
** **Knew a place to test for HIV	1.7 (1.12–2.51)*	3.9 (1.02–14.9)*
** **Ever tested for HIV	1.8 (1.13–2.97)**	7.3 (2.6–20.7)***
** **Use of SRH service (past three months)	0.8 (0.55–1.16)	3.2 (1.62–6.27)***

All analyses were adjusted for age, education, religion and types of work.

* Significant at *p<*0.05

** significant at *p<*0.01

*** significant at *p<*0.001. AOR, adjusted odds ratio comparing end line to baseline; CI, confidence interval; SRH, sexual and reproductive health.

## Discussion

This is the first study to pilot test and assess the effects of a targeted psychosocial intervention among a migrant, adolescent population in Ethiopia. Our study suggests that a psychosocial counselling intervention was associated with increased knowledge and uptake of HIV and sexual health services among both male and female vulnerable adolescents, as well as reduced mental health problems among female adolescents. In particular, the intervention was associated with increased HIV prevention knowledge and HIV testing for both male and female adolescents, as well as increased use of SRH services among males. Although the intervention was associated with reduced aggressive behaviours and overall mental health problems among females, there was no effect on mental health indicators among male participants.

Available studies conducted in small populations across Ethiopia show a wide-ranging prevalence of mental health problems, varying from 5.5% [28] to 49% of non-at-risk youth reporting mental disorders or mental distress [29]. A key message is that it appears that separation from family is associated with higher levels of mental health problems among young people [30]. Our findings are consistent with findings from the limited available studies in African settings in which psychosocial interventions also resulted in desired behavioural outcomes, but results were mixed for reducing mental health problems [31–33].

A randomized control trial evaluating a school readiness programme with male and female child soldiers in Sierra Leone included many of the same intervention components used in this study, such as addressing mental health stresses and risky behaviours. It found that the youth readiness intervention programme was associated with improved school readiness behaviours but no change in underlying psychological and emotional problems [32]. Other studies among high-risk African-American female adolescents also found that various psychosocial and health promotive group interventions resulted in improved knowledge and behaviour for HIV prevention [31,33].

A related study in South Africa using a community-based art therapy intervention among HIV-affected children and adolescents (ages 8 to 18) found no significant reduction of depression or emotional and behavioural problems, but it did find that the intervention significantly increased participants’ sense of self-worth and self-efficacy for decision making and dealing with a difficult environment [34]. Furthermore, a trauma-focused cognitive behaviour therapy intervention implemented with female adolescents in the Democratic Republic of Congo, who had been sexually exploited and war affected, found significant reduction in psychological distress and psychosocial difficulties [35].

Taken together, these findings suggest that, although behavioural changes may be detected after a short duration of intervention, changes in psychosocial and mental health problems will likely require an intervention period longer than three months to show any detectable changes.

The differences in use of sexual health services (including seeking counselling for safer sex and HIV-prevention methods) between females and males could be attributed to the accessibility of HIV testing and health education services. Biruh Tesfa mentored females were referred to government-run clinics for health information and services, including HIV testing, whereas males accessed HIV testing and SRH services on site at Retrak with a staff nurse who was known to them. This familiarity of the staff and ease of access may have accounted for an increased use of SRH services among males.

Our findings indicate that a psychosocial intervention addressing underlying mental health issues might foster vulnerable adolescents’ capacity to benefit from HIV risk-reduction education and use of HIV-related services. Using a client-centred approach in a targeted psychosocial intervention may improve the psychosocial wellbeing and self-efficacy of participants, ultimately facilitating behaviour change.

The reduction in mental health problems seen only among female adolescents may be explained as follows. First, there were important differences in the groups of male and female adolescents. For example, the prevalence of mental health problems of all four indicators was much higher among males than among females at baseline, suggesting that the intervention might have to be gender specific to see a significant impact.

Second, males were living in more difficult situations – all had previously lived or worked on the street, possibly making their mental health problems more sustained or difficult to change. In addition, they may have required a more intensive, tailored or lengthier psychosocial intervention in order to see a significant change in mental health outcomes.

Finally, Retrak staff reported that when male adolescents first joined the programme, they were quiet and withdrawn, but as they developed trust in Retrak staff and felt safe, they expressed their anger and frustration and became much more demanding and aggressive, which often lasted until they were reintegrated with their families or society. This could partially explain the insignificant mental health findings among male participants.

### Limitations

It is possible that sexual activity and/or sexual abuse data were underreported among this population due to social desirability or recall bias, especially in the context of a face-to-face interview.

In addition, the study did not have a control group due to the inability to recruit a larger population for an experimental study design. This limited our ability to tease out the true impact of the intervention on key outcomes. However, this approach is particularly acceptable because the study was designed as operational research with the aim of pilot testing the intervention and measuring changes in outcomes among participants. Resources permitting, a follow-up controlled study would be ideal for examining the impact of the intervention.

Moreover, the study had a high rate of LFU, especially among female participants. LFU could have reduced the statistical power in detecting significant changes in key outcomes. The high LFU rate was due to the mobility of this population and time and financial constraints, which impacted the number of mentors who remained available to assist study counsellors to follow up with study participants for subsequent counselling sessions. Lastly, due to financial and logistical constraints and the high mobility of this migrant population, the intervention could not be delivered for longer than a three-month period.

## Conclusions

The findings suggest that a psychosocial intervention is associated with increased knowledge and uptake of HIV and sexual health services among both male and female migrant adolescents, and with reduced mental health problems among female adolescents. The mental health problems of male and female adolescents varied significantly, suggesting that future interventions should be tailored to address their different needs. Furthermore, future operational research using a controlled design among migrant adolescents is needed and would benefit from intensive follow-up efforts to reduce LFU or from a larger sample size.
